# A multicenter open-label phase II trial to evaluate nivolumab and ipilimumab for 2nd line therapy in elderly patients with advanced esophageal squamous cell cancer (RAMONA)

**DOI:** 10.1186/s12885-019-5446-2

**Published:** 2019-03-14

**Authors:** Nadja M. Meindl-Beinker, Johannes Betge, Tobias Gutting, Elke Burgermeister, Sebastian Belle, Tianzuo Zhan, Nadine Schulte, Martin Maenz, Matthias P. Ebert, Nicolai Haertel

**Affiliations:** 1 Department of Medicine II, University Hospital Mannheim, Medical Faculty Mannheim, Heidelberg University, Theodor-Kutzer-Ufer 1-3, 68167 Mannheim, Germany; 2 AIO-Studien gGmbH, Berlin, Germany

**Keywords:** Esophageal squamous cell cancer, Elderly, Comprehensive geriatric assessment, Checkpoint inhibitors, Personalized medicine, Geriatric oncology

## Abstract

**Background:**

Advanced esophageal squamous cell cancer (ESCC) is frequently diagnosed in elderly patients. The impact of 2nd line chemotherapy is poorly defined. Recent data demonstrated effectiveness of checkpoint inhibitors in different squamous cell carcinomas. Therefore, we assess combined nivolumab/ipilimumab as 2nd line therapy in elderly ESCC patients.

**Methods:**

RAMONA is a multicenter open-label phase II trial. The primary objective is to demonstrate a significant survival benefit of nivolumab/ipilimumab in advanced ESCC compared to historical data of standard chemotherapy. Primary endpoint is therefore overall survival (OS). Major secondary objective is the evaluation of tolerability. Time to QoL deterioration will thus be determined as key secondary endpoint. Further secondary endpoints are tumor response, PFS and safety. We aim to recruit a total of *n* = 75 subjects that have to be > 65 years old. Eligibility is determined by the geriatric status (G8 screening and Deficit Accumulation Frailty Index (DAFI)). A safety assessment will be performed after a 3 cycle run-in phase of nivolumab (240 mg Q2W) to justify escalation for eligible patients to combined nivolumab (240 mg Q2W) and ipilimumab (1 mg/kg Q6W), while the other patients will remain on nivolumab only. RAMONA also includes translational research sub-studies to identify predictive biomarkers, including PD-1 and PD-L1 evaluation at different time points, establishment of organoid cultures and microbiome analyses for response prediction.

**Discussion:**

The RAMONA trial aims to implement checkpoint inhibitors for elderly patients with advanced ESCC as second line therapy. Novel biomarkers for checkpoint-inhibitor response are analyzed in extensive translational sub-studies.

**Trial registration:**

EudraCT Number: 2017–002056-86; NCT03416244, registered: 31.1.2018.

**Electronic supplementary material:**

The online version of this article (10.1186/s12885-019-5446-2) contains supplementary material, which is available to authorized users.

## Background

ESCC is the sixth leading cause of cancer-related death worldwide [1]. The disease is frequently diagnosed in advanced tumor stages and in elderly patients [1, 2].

Efficacy of chemotherapy in advanced ESCC is still poorly defined. While most patients undergo chemotherapy and/or chemo-radiation in first line according to the CROSS protocol using Paclitaxel and Carboplatin, effectiveness of second-line chemotherapy is discouraging [3, 4]. However, very recently Kojima et al. reported that pembrolizumab significantly improved OS compared to chemotherapy (paclitaxel, docetaxel or irinotecan) in patients with advanced esophageal or esophagogastric junction carcinoma whose tumors express PD-L1 (Combined Positive Score [CPS] ≥10, regardless of histology) (median 9.3 vs 6.7 mo; HR 0.69; 95% CI 0.52–0.93; *P* = 0.0074). OS at 12 months was 43% vs 20%, respectively. (KEYNOTE 181) [5].

Immunotherapy with antibodies against immune checkpoints like PD-1/PD-L1 represents a new treatment opportunity with relatively little side effects and first promising results in the treatment of squamous cell carcinoma patients [6–8]. With respect to esophageal cancer, preliminary results from an Asian study indicate efficacy of nivolumab [9]. From 64 heavily pre-treated patients, 11 (17, 95% CI 10–28) had an objective response and 16 (25, 95% CI 16–37) demonstrated stable disease. The median overall survival was 10.8 months (IQR 4.9–14.3) in this trial population (unselected for PD-L1 expression status). Long-term survival was also improved by pembrolizumab as described by Doi et al. [10].

Furthermore, the CheckMate 012 trial demonstrated that overall response rates could be doubled when PD-1 inhibitor nivolumab was combined with CTLA-4-inhibitor ipilimumab in advanced NSCLC patients [11]. In this trial, grade 3 and 4 adverse events were reported to occur in 33% of the patients treated with the combination therapy (nivolumab 3 mg/kg Q2W and ipilimumab 1 mg/kg Q6W). In the checkmate 032 in turn (nivolumab 3 mg/kg Q2W and ipilimumab 1 mg/kg Q3W), treatment related adverse events of grade 3 and 4 were only slightly enhanced when compared to nivolumab monotherapy (13% vs. 19%) [12].

There is an increasing need for improved treatment strategies for elderly ESCC patients. These strategies have to acknowledge the challenges of functional limitations and comorbidities in this increasing population. With increasing age, elderly patients develop chronic diseases and different comorbidities that may affect person’s capabilities, functional reserve and life expectancy [13]. However, assessment of these characteristics in the elderly population is time-consuming, therefore new assessment and screening tools are being developed. The poor knowledge of the role of chemotherapy and immunotherapy in these individuals, due to lack of enrolment of these patients in clinical trials, demands for novel concepts of clinical trials specifically designed for elderly patients.

With the RAMONA trial, we aim to address this high medical need by assessing nivolumab and ipilimumab in combination as second-line therapy of advanced ESCC in the elderly population. Patient eligibility will be assessed by geriatric screening tools in this trial. Moreover, we aim to establish novel biomarkers for checkpoint inhibition by extensive translational sub-studies.

## Methods/design

### Study design

RAMONA is a multicenter open-label phase II trial conducted in 34 centers in Germany (for overviews please refer to Additional file 1: Table S1). Key inclusion criteria are

- age ≥ 65 years at time of recruitment
- histologically approved diagnosis of advanced ESCC
- progression after front line treatment (including chemo-radiation with carboplatin/paclitaxel or others)

A screening phase is used to determine the eligibility of a patient and may last up to 4 weeks before initiation of treatment. Eligibility and the geriatric status of potential patients will be assessed using the G8 screening tool and the Deficit Accumulation Frailty Index (DAFI). Patients are recruited independent of their PD-1 or PD-L1 expression status.

The dosing rationale for the study is based on Kudo et al. [9] and Hellmann et al. [11]. The treatment phase begins with 2nd-line nivolumab monotherapy safety run-in (240 mg fixed dose Q2W). Based on reports by Kähler et al., [14, 15] a safety assessment will be performed after 3 cycles of nivolumab monotherapy (6 weeks), during which the investigator will decide if a patient is eligible to receive nivolumab/ipilimumab combination therapy (Nivo 240 mg fixed dose Q2W; ipilimumab 1 mg/kg Q6W; arm A) or continues nivolumab monotherapy (240 mg fixed dose Q2W; arm B). This safety assessment was added due to the substantial comorbidities in most ESCC patients which may increase adverse events as compared to patients with other carcinomas and which might e.g. counteract treatment escalation. Only those patients with toxicities grade ≤ 2 will be escalated. In both arms, treatment continues until progressive disease or intolerable toxicity or withdrawal of consent or death. After another 3 cycles of treatment in both arms (approx.12 weeks from first dose) a restaging examination by radiologic imaging will be performed, which is repeated every four treatment cycles thereafter. Treatment within the context of the study is limited to 2 years.

**Fig. 1:**
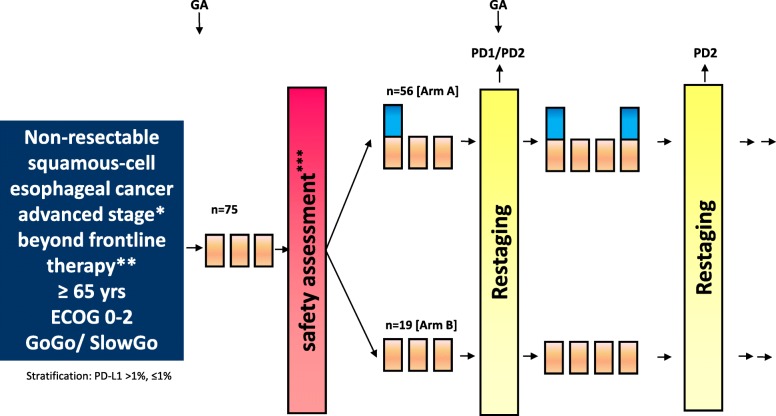
Study scheme. Patients may be escalated to nivolumab/ipilimumab combination therapy after a 3 cycle nivolumab-only run-in phase and positive safety assessment. GA: geriatric assessment (G8 / DAFI). ***: treatment escalation according to safety assessment results (please see full protocol). **: Chemotherapy (+/− radiotherapy) (e.g. CROSS, FLOT or similar protocols) OR any palliative systemic chemotherapy. * Stage 4 OR stage 3 non-responder to radio-chemotherapy OR any relapse after chemo-radiation OR any relapse after surgery if patient is ineligible or intolerant to standard frontline therapy OR refuses other treatment. PD1: off study despite post-progression ipi/nivo in case of toxicity and/or clinical deterioration. PD2: off study if confirmed progression according to Recist criteria V 1.1 or non-response according to immune-related response criteria. Ipilimumab 1 mg/kg IV Q6W. Nivolumab 240 mg IV Q2W. Restaging (incl. Endoscopic biopsies +PD-L1 staining)

Samples for translational research are gathered at different time points, i.e. screening/baseline (no therapy), safety assessment (after 3 cycles of nivolumab monotherapy) and restaging (after 3 cycles in treatment arm A or arm B). The study design is illustrated in Fig. 1.

### Study objectives

The primary objective of this trial is to demonstrate a significant survival benefit of the combination therapy with nivolumab/ipilimumab treatment in advanced ESCC compared to historical data of standard chemotherapy regimens [4].

Secondary objectives:

Tolerability of nivolumab as single agent and in combination with ipilimumab will be investigated in terms of QoL. Hence, a key secondary endpoint ‘time to QoL deterioration’ will be implemented.

Further secondary objectives are the assessment of additional efficacy and safety parameters of an intensified immunotherapy regimen and the assessment and exploration of the predictive value of structured geriatric assessments for treatment-emergent toxicities and treatment discontinuation.

### Measurements

For patient screening, the G8 screening tool and the Deficit Accumulation Frailty Index (DAFI) will be used. Patients with G8 score > 14 will be stratified as non-frail and can be directly included into to study (GoGo). If G8 scores ≤14 (frail), the DAFI questionnaire needs to be performed. DAFI-indices between 0.2 and 0.35 qualify patients as SlowGo enabling the PI to decide whether a patient is eligible or cannot participate. DAFI index ≥0.35 is defined as exclusion criterion.

In terms of safety, the screening includes an intensified assessment of cardiac co-morbidities using echocardiogram and measurement of cardiac enzymes (e.g. troponin-I). QoL will be assessed by the EORTC questionnaires QLQ-C30 and ELD14.

### Safety assessment

Procedures to be conducted during the treatment phase of the study include a safety assessment after 3 weeks (3 cycles) of nivolumab monotherapy. A study subject is eligible to escalate to nivolumab/ipilimumab combination therapy if I) the study subject has sufficient cardiac functional reserve determined by cardiac echo (less than 10% decline in ejection fraction compared to baseline assessment), II) no clinically significant abnormal troponin or ECG and III) the study subject is potentially benefitting from treatment escalation according to the judgment of the investigator.

### End of treatment

All subjects will be followed for survival until the end of the study, regardless of further treatments, or until the sponsor ends the study (follow-up extension phase).

The following procedures will be performed during follow-up every 6 weeks for the first year of follow-up, and every 8–12 weeks thereafter:

- assessment of survival status
- recording of all anti-cancer treatments
- recording of all AEs and SAEs for 100 days after last dosing

### Primary and secondary end points

#### Primary endpoint

- Overall survival

#### Secondary endpoints

Key secondary endpoint:

- Time to QoL deterioration defined as a loss of ≥10 points in the EORTC QLQ-C30 compared to base-line

Additional secondary endpoints:

- PFS
- ORR according to RECIST 1.1 and immune related response criteria (modified RECIST)
- Duration of Response (DOR)
- Duration of treatment
- cumulative dose intensity
- QoL (EORTC QLQC30 and ELD14)
- AEs/SAEs, toxicities according to CTC criteria v4.0
- Geriatric assessments:
  - Evaluation of the predictive value of the GA containing tests (DAFI, G8-Questionaire etc.) for the occurrence of ≥ grade 3 toxicities
  - Predictive value of the assessed geriatric tests for treatment discontinuation
- Translational research at different time points (screening, safety assessment and first restaging)
- PD-1-PD-L1 expression in tumor tissue before and during treatment
- predictive biomarkers in tumor tissue (pre-treatment and re-biopsies) and blood
- establishment of organoid cultures from tumor tissue specimens
- Response prediction by microbiome assessment

### Study setting

Patients will be recruited at 34 selected study sites in Germany including academic hospitals, community clinics and practitioner to achieve adequate participant enrolment. A list of study sites can be obtained from the corresponding author of this manuscript. Protocol modifications will be communicated to relevant parties upon ethics approval.

### Data collection

Data for this study will be recorded via eCRF by the site from the source documents according to standard operational procedures. Data are reviewed and checked for omissions, apparent errors, and values requiring further clarifications using computerized (automatic) and/or manual procedures. Accurate and reliable data collection will be assured by verification and cross–check of the eCRF against the investigator’s records by the study monitor. Data will be recorded and reported until the last subject will have completed the trial. Informed consent or assent forms including additional informed consent/assent forms for participation in the RAMONA translational research program will remain at the respective study sites. Confidentiality of personal information is protected by adherence to the European GDPR.

### Statistical analysis and sample size

It is hypothesized that nivolumab and ipilimumab will increase overall survival. It is assumed that an immunotherapy approach consisting of a nivolumab monotherapy in conjunction with a safety guided treatment escalation to a nivolumab/ipilimumab combination regimen increases the 1-year overall survival rate by a margin of 13% compared to historical control for standard chemotherapy (i.e. nivolumab monotherapy followed by a conditional nivolumab + ipilimumab therapy 1-yr-OS = 30% vs CTx-control 1-yr-OS = 17%) [4]. Based on these assumptions, and an exponential shape of the survival curve, a one-sided, one-sample log rank test calculated from a sample of 69 subjects achieves 90.3% power at an alpha = 0.05 one-sided significance level to detect a proportion surviving of 0.3 in the experimental group when the proportion surviving in the historic control group is 0.17. These proportions surviving are for a period of 12 month (1-year-OS rate). Subjects are accrued for a period of 12 month. Follow-up continues for a period of 24 month after the last subject is added. The probability that a subject experiences an event during the study is 0.9477. The expected number of events during the study is 65. To compensate for uninformative drop-outs a total of *N* = 75 subjects need to be recruited. All patients, both on monotherapy and on combination therapy, are subject to statistical ITT analysis.

## Discussion

In the CROSS trial, van Hagen et al. demonstrated that first-line neoadjuvant chemoradiation using carboplatin and paclitaxel is a highly effective regimen for locally advanced ESCC, leading to patients´ overall survival of more than 50 months in single cases (median DOR of 15 months) [3]. This approach is also considered effective as definite chemoradiotherapy for advanced ESCC patients who are not candidates for subsequent surgery [16] and therefore commonly used in Germany. In these patients, second line treatment with paclitaxel is not an option. However, treatment options after chemoradiation with platin derivatives and taxanes for patients with recurrent cancers are limited. Thallinger and coworkers convincingly demonstrated that 2nd line therapy for these patients is un-standardized [4]. Clinical trials focusing on 2nd line chemotherapy for ESCC patients showed frustrating response rates, enhanced toxicity rates and poor overall survival, mostly limited to 6–8 months [4]. Furthermore, majority of ESCC patients are elder persons [17] with significant co-morbidities which in the clinical practice typically renders these patients ineligible for poly-chemotherapy.

To address this issue, the aim of the RAMONA trial is to evaluate a promising new second line therapy option for elderly ESCC patients. Nivolumab as immunotherapeutical agent blocks interaction of PD-1 molecules expressed by tumor-specific T-cells with its ligand PD-L1 on tumor cells, as reviewed in [18]. PD-L1 expression seems to be significantly increased in ESCC with age and is associated with poor prognosis [19]. Pooled analysis of CheckMate 017 and checkmate 057 studies demonstrated, that higher PD-L1 expression levels were associated with greater OS benefit with nivolumab (HR, 0.42; 95% CI, 0.28 to 0.63) in patients with ≥50% PD-L1 expression, but a benefit was still observed in patients with < 1% PD-L1 expression (HR, 0.78; 95% CI, 0.61 to 0.99) [20]. However, here effects seem to more prominent in non-squamous NSCLC. Antonia and colleagues very recently presented new data hinting towards improved ORR in correlation with PDL-1 expression levels upon nivolumab/ipilimumab combination therapy as compared to nivolumab monotherapy (Chicago Multidisciplinary Symposium in Thoracic Oncology, 2016, [11]). Despite further promising results indicating a correlation of good ORR with expression of PD-L1 in lung cancer (PD1 expression ≥1% ORR 57% vs. 28%) [11, 21], the predictive value of PD-1/PD-L1 expression for nivolumab-based therapies is not fully understood in most tumor entities. Therefore, we implemented a translational part into the trial monitoring PD-1/PD-L1 expression rates during the course of treatment.

Checkpoint-inhibitor treatments combine several advantages, which are especially interesting with regard to ESCC treatment approaches. Firstly, promising overall survival has been reported in squamous cell carcinoma patients of different entities, pointing towards a rationale to test efficacy also in ESCC [6, 11, 22–24]. Secondly, antitumor activity of checkpoint inhibitors was shown when used after radiation-based treatments, which is commonly also performed in ESCC treatment. For instance, in a randomized phase III trial including stage II NSCLC patients (i.e. patient that undergo surgery with curative intent), Antonia and coworkers demonstrated that PFS was increased by durvalumab treatment after chemotherapy/radiotherapy compared to chemotherapy/radiotherapy alone [25]. In line, NSCLC patients treated with pembrolizumab showed longer PFS (HR 0.56 [95% CI 0.34–0.91], *p* = 0.019) and increased OS (HR 0.58 [95% CI 0.36–0.94], *p* = 0.026) if they had received any radiotherapy as compared to non-radiated patients [26]. An association of radiotherapy with improved rates of index lesion response in 68% of melanoma patients treated with ipilimumab [27] further underline possible synergistic effects of radiation and immunotherapy on local on distant tumor control as reviewed in [28].

Kudo et al. reported that even for heavily pretreated ESCC patients, immunotherapy with nivolumab alone prolonged overall survival of Asian patients by median 10.8 months (95% CI 7·4–13·3) [9]. Additionally, the risk profile of ESCC patients [1, 29–32] in part overlaps with that of NSCLC patients who are often heavy smokers. In line, available data reveals that especially heavy smokers seem to profit from immunotherapy (Objective response: current and former smokers vs never-smokers (46% [30 of 65] vs 27% [3 of 11]) [11].

ESCC is most frequently diagnosed between 65 and 74 years [17], defining the patient cohort as mostly elderly with per se accumulated co-morbidities, impaired pharmacokinetics and a potentially declined functional reserve [13]. To objectify the functional reserve of the patients and its impact on treatment outcome, a comprehensive geriatric assessment (CGA) is recommended [33]. However, CGA is time consuming and rarely feasible in clinical practice. Thus, we decided to assess the vulnerability of our study subjects by the G8 questionnaire and implement the more complex DAFI assessment only for patients below G8 cut-off (≤14 points). The DAFI is a condensed, validated CGA with 51 items lasting approximately 30 min of which > 80% can be self-reported by the patient [34]. Based on Cohen et al., we estimated that ~ 40% of the patients will belong to a pre-frail group with DAFI indices between 0.2 and 0.35. These patients can enter the RAMONA study upon investigator’s decision as immune-oncological treatments are known to be well-tolerated [11]. Even for combined nivolumab/ipilimumab therapy, grade ≥ 3 toxicities are reported to occur in only 33% in NSCLC [11]. As the CheckMate Trial 214 revealed grade 3 and 4 toxicities in up to 46% of the patients with advanced renal cell carcinoma treated with nivolumab and ipilimumab [35], we consider the RAMONA dosage treatment scheme with longer intervals of ipilimumab administration to provide better tolerability (CheckMate 214 ipilimumab Q3W as compared to Q6W in our study).

However, to ensure that patients are eligible for nivolumab/ipilimumab combination therapy in the RAMONA trial, a safety assessment is performed after a 3 week run-in phase of nivolumab alone. Only those patients with toxicities grade ≤ 2 will be escalated.

Taken together, we used the findings discussed above to formulate the rationale of the RAMONA study and to transfer current knowledge on immunotherapy to the fragile cohort of elderly Caucasian ESCC patients. Overall survival was selected as primary endpoint. In line, impressive long term responses of immunotherapy (at least with pembrolizumab) of up to 50 months (median time to initial response of 4 months (range, 2 to 8 months); median DOR of 15 months (range, 6 to ≥26 months) are reported, even after end of treatment [10]. Treatment adherence is often negatively impacted by quality of life (QoL) deterioration in cancer patients. Therefore, adequate preservation of QoL is an important clinical goal in in elderly patients with recurrent ESCC. Thus, ‘Time to QoL Deterioration’ was included as a key secondary endpoint in this trial.

## Conclusion

In conclusion, RAMONA is the first trial to evaluate checkpoint inhibitor based immunotherapy as a novel treatment opportunity in elderly ESCC patients. Based on a recruitment period of 12 months, first results are expected end of 2019.

### Trial status

RAMONA is an ongoing study. Recruitment commenced in April 2018 and the study is at the moment recruiting patients. For a status update please refer to https://clinicaltrials.gov/ct2/show/NCT03416244.

## Additional file

Additional file 1: **Table S1.** RAMONA. Title of data: RAMONA synopsis. Description of data: synopsis of the RAMONA trial. (DOCX 37 kb)

## Abbreviations

AEs/SAEs: Adverse events, serious adverse events
CGA: Comprehensive geriatric assessments
CI: Confidence interval
CTC: Common toxicity criteria
CTLA-4: Cytotoxic T-lymphocyte-associated Protein 4
DAFI: Deficit accumulation frailty index
DOR: Duration of Response
ECOG: Eastern Cooperative Oncology Group
ESCC: Esophageal squamous cell cancer
HR: Hazard ratio
ORR: Objective response rate
OS: Overall survival
PD-1, PD-L1: Programmed cell death protein 1, Programmed death-ligand 1
PFS: Progression-free survival
QLQ-C30: Quality of life questionnaire C30
QLQ-ELD14: Quality of life questionnaire ELD14
QoL: Quality of life
RECIST: Response Evaluation Criteria In Solid Tumors
