# Supplementary material for: A multicenter open-label phase II trial to evaluate nivolumab and ipilimumab for 2nd line therapy in elderly patients with advanced esophageal squamous cell cancer (RAMONA)
Source: BMC Cancer. 2019 Mar 14;19:231. doi: 10.1186/s12885-019-5446-2 (PMC6419339; doi:10.1186/s12885-019-5446-2)
Supplement: Supplementary file 1 — Table S1. RAMONA. Title of data: RAMONA synopsis. Description of data: synopsis of the RAMONA trial. (DOCX 37 kb) [file 12885_2019_5446_MOESM1_ESM.docx]

| Title | A multicente**R** open-label phase II tri**A**l to evaluate Nivolu**M**ab and Ipilimumab f**O**r 2nd line therapy in elderly patie**N**ts with advanced esophageal squamous cell c**A**ncer |
| --- | --- |
| Acronym | RAMONA |
| Protocol Version | V3.0 |
| Primary registry and trial identifying number | NCT03416244 |
| Date of registration in primary registry | 31.1.2018 |
| Secondary identifying numbers | EudraCT Number: 2017-002056-86, AIO-STO-0117 |
| Sponsor and contact for public queries | AIO-Studien-gGmbH  Dr. Aysun Karatas, Kuno-Fischer-Straße 8, 14057 Berlin  Phone: +49-30 – 8145 344 31  Fax: +49-30 – 3229 329 26  E-Mail: info@aio-studien-ggmbh.de |
| Source(s) of monetary or material support | Bristol-Myers Squibb GmbH & Co. KGaA |
| Principal investigator and contact for scientific queries | Prof. Dr. med. Matthias Ebert  Universitätsmedizin Mannheim  Theodor-Kutzer-Ufer 1-3  68167 Mannheim, Germany  Phone: +49621 383 3284  Fax: +49621 383 3905  E-Mail: Matthias.Ebert@umm.de |
| Data monitoring | AIO-Studien-gGmbH |
| Safety desk | AIO-Studien-gGmbH |
| Anticipated start date | Q4/2017 |
| Duration of study | Enrollment: 12 month  total study duration 36 month (incl. follow-up) |
| Indication | Pretreated advanced esophageal squamous cell cancer (ESCC) |
| Total number of sites | 34 sites in Germany |
| Primary objective | The primary objective of this trial is to demonstrate a significant  survival benefit of the combination therapy with Nivolumab/Ipilimumab treatment in advanced esophageal squamous cell cancer compared to historical data of standard chemotherapy regimens.  Additionally, tolerability of Nivolumab as single agent and in combination with Ipilimumab will be investigated in terms of quality of life. Hence, a key secondary endpoint ‘time to QoL deterioration’ will be implemented. |
| Secondary objectives | Secondary objectives of this study are:   - to assess additional efficacy and safety parameters of an intensified immunotherapy regimen. - to assess and explore the predictive value of structured geriatric assessments for treatment-emergent toxicities and treatment discontinuation. |
| Planned sample size | **N=75 enrolled to receive Nivolumab monotherapy followed by**  **Nivolumab/Ipilimumab combination therapy conditional upon**  **tolerablitity** |
| Inclusion Criteria | 1. Written informed consent including participation in translational research and any locally-required authorization (EU Data Privacy Directive in the EU) obtained from the subject prior to performing any protocol-related procedures, including screening evaluations  2. Age ≥ 65 years at time of study entry  3. Histologically confirmed advanced stage non-resectable  esophageal squamous cell carcinoma beyond frontline therapy*:  • stage 4 OR  • stage 3 non-responder to radio-chemotherapy OR  • any relapse after chemo-radiation OR  • any relapse after surgery if patient is ineligible or intolerant to standard frontline therapies OR refuses other treatment  * Frontline therapy is defined as chemotherapy (+/- radiotherapy) (e.g. CROSS, FLOT or similar protocols) OR any palliative systemic chemotherapy  4.Geriatric status: SlowGo or GoGo according to G8 and DAFI assessment (G8 > 14 points or CGA/DAFI 0.2 < 0.35)  5. At least 1 measurable lesion according to RECIST 1.1  6. Karnofsky performance status ≥ 50  7. Sufficient cardiac functional reserve defined as ejection fraction ≥ 50%  8. Adequate blood count, liver-enzymes and renal function:   - neutrophil count > 1.5 x 106/mL   WBC ≥ 3000/μL   - Platelet count ≥ 100 x 109/L (> 100,000 per mm3) - hemoglobin ≥ 9 g/dL - INR ≤ 1.5 and PTT ≤ 1.5 x ULN during the last 7 days before therapy - AST (SGOT)/ALT (SGPT) < 3 x institutional upper limit of normal (5 x lower limit in case of liver metastases) - bilirubin < 1.5 x ULN - Serum creatinine ≤ 1.5 x institutional ULN or creatinine clearance (CrCl) ≥ 30 mL/min (if using the Cockcroft-Gault formula below):   Female CrCl = (140 - age in years) x weight in kg x 0.85  72 x serum creatinine in mg/dL  Male CrCl = (140 - age in years) x weight in kg x 1.00  72 x serum creatinine in mg/dL  9. Men who are sexually active with WOCBP must use any contraceptive method with a failure rate of less than 1% per year. Men receiving Nivolumab and who are sexually active with WOCBP will be instructed to adhere to contraception for a period of 7 months after the last dose of investigational products (Nivolumab, Ipilimumab). Women who are not of childbearing potential (i.e., who are postmenopausal or surgically sterile) as well as azoospermic men do not require contraception)  10. Subject is willing and able to comply with the protocol for the  duration of the study including undergoing treatment and scheduled visits and examinations including follow up |
| Exclusion Criteria | **Methodological criteria:**  1. Patients < 65 years of age  2. Frail patients (DAFI score ≥ 0.35)  3. Esophageal adenocarcinomas, neuroendocrine tumors  4. Prior therapy with an anti-programmed cell death protein 1 (anti-PD-1), anti-PD-L1, anti-programmed cell death-ligand 2 (anti-PD-L2), anti-CD137 (4-1BB ligand, a member of the Tumor Necrosis Factor Receptor [TNFR] family), or anticytotoxic T-lymphocyte-associated antigen-4 (anti-CTLA-4) antibody (including Ipilimumab or any other antibody or drug specifically targeting T-cell co-stimulation or checkpoint pathways)  5. Participation in another clinical study with an investigational product during the last 30 days before inclusion or 7 half-lifes of previously used trial medication, whichever is longer  6. Previous treatment in the present study (does not include screening failure).  **Medical:**  7. Any condition or comorbidity that, in the opinion of the investigator, would interfere with evaluation of study treatment or interpretation of patient safety or study results, including but not limited to:  a) Major surgery ≤ 28 days prior first dose of study treatment  b) Anticancer treatment during the last 30 days prior to start of Nivolumab monotherapy treatment, including systemic therapy, or major surgery [palliative radiotherapy has to be completed at least 2 weeks prior to start of study treatment]  c) History of interstitial lung disease  d) Known acute or chronic pancreatitis  e) Known active HBV, HCV or HIV infection  f) Active tuberculosis  g) Any other active infection (viral, fungal or bacterial) requiring systemic therapy  h) History of allogeneic tissue/solid organ transplant  i) Diagnosis of immunodeficiency or patient is receiving chronic systemic steroid therapy or any other form of immunosuppressive therapy within 7 days prior to the first dose of Nivolumab monotherapy treatment.  j) Has an active autoimmune disease requiring systemic treatment within the past 3 months or a documented history of clinically severe autoimmune disease or a syndrome that requires systemic steroids or immunosuppressive agents.  k) Live vaccine within 30 days prior to the first dose of Nivolumab monotherapy treatment or during study treatment.  l) Other clinically significant active malignancy requiring treatment OR less than 5 years disease free interval of another primary malignancy  m) Clinically significant or symptomatic cardiovascular/cerebrovascular disease (incl. myocardia infarction, unstable angina, symptomatic congestive heart failure, serious uncontrolled cardiac arrhythmia) within months before enrollment  n) History or clinical evidence of CNS metastases; Exceptions are: Subjects who have completed local therapy and who meet both of the following criteria:  I. are asymptomatic AND  II. have no requirement for steroids 6 weeks prior to start of Nivolumab monotherapy treament. Screening with CNS imaging (CT or MRI) is required only if clinically indicated or if the subject has a history of CNS metastases  **Drug related criteria:**  8. Medication that is known to interfere with any of the agents applied in the trial.  9. Has known hypersensitivity to Nivolumab or Ipilimumab or any of the constituents of the products  10. Any other efficacious cancer treatment except protocol specified treatment at study start  11. Patient has received any other investigational product within 28 days of study entry  **Safety criteria:**  12. Patient has had a prior monoclonal antibody within 4 weeks prior to study Day 1 or who has not recovered (i.e., ≤ Grade 1 or at baseline) from adverse events due to agents administered more than 4 weeks earlier. [Subjects with ≤ Grade 2 neuropathy or alopecia are an exception to this criterion and may qualify for the study.]  13. Female subjects who are pregnant, breast-feeding or male/female patients of reproductive potential who are not employing an effective method of birth control (failure rate of less than 1% per year). [Acceptable methods of contraception are: implants, injectable contraceptives, combined oral contraceptives, intrauterine pessars (only hormonal devices), sexual abstinence or vasectomy of the partner]. Women of childbearing potential must have a negative pregnancy test (serum β-HCG) at screening.  **Regulatory and ethical criteria:**  14. Patient with any significant history of non-compliance to medical regimens or with inability to grant reliable informed consent  15. Patient who has been incarcerated or involuntarily institutionalized by court order or by the authorities § 40 Abs. 1 S. 3 Nr. 4 AMG  16. Patients who are unable to consent because they do not understand the nature, significance and implications of the clinical trial and therefore cannot form a rational intention in the light of the facts [§ 40 Abs. 1 S. 3 Nr. 3a AMG] |
| Investigational Medicinal Product | - Nivolumab - Ipilimumab |
| Treatment schedule | Subjects enrolled in this trial will initiate 2nd line palliative systemic treatment with Nivolumab monotherapy (240 mg Q2W) for 3 consecutive cycles (safety run-in). After three cycles of Nivolumab monotherapy study subjects will be assessed for the occurrence of specific treatment-emergent adverse events (TEAE).  Study subjects without significant TEAEs are eligible to escalate  treatment to a Nivolumab/Ipilimumab combination therapy:  **Arm A: Nivolumab 240 mg fixed dose IV Q2W; Ipilimumab 1mg/kg IV Q6W (starting in week 7 after safety assessment)**  Subjects with significant TEAEs who still qualify for Nivolumab treatment continue Nivolumab monotherapy:  **Arm B: Nivolumab 240 mg IV fixed dose Q2W**  In both arms treatment continues until progressive disease or intolerable toxicity or withdrawal of consent or death. Treatment within the context of the study is limited to 2 years.  Tumor assesssments:   - 1st restaging assessment after 12 weeks of therapy - thereafter Q8W (applies equally to any confirmatory assessments according to RECIST 1.1 or modified RECIST as well as any re-evaluation of treatment beyond progression) |
| Primary endpoint | Overall survival |
| Secondary endpoints | Key secondary endpoint:   - Time to QoL deterioration defined as a loss of ≥ 10 points in the EORTC QLQ-C30 compared to base-line   Additional secondary endpoints:   - PFS - ORR according to RECIST 1.1 and immune related response - criteria (modified RECIST) - Duration of Response (DOR) - Duration of treatment - cumulative dose intensity - QoL (EORTC QLQC30 and ELD14) - AEs/SAEs   Geriatric assessments:   - Evaluation of the predictive value of the GA containing tests (DAFI, G8-Questionaire etc.) for the occurrence of ≥ grade 3 toxicities - Predictive value of the assessed geriatric tests for treatment discontinuation |
| Translational  research:  Exploratory  objectives and  endpoints | - predictive biomarkers in tumor tissue (pre-treatment and rebiopsies) and blood - establishment of organoid cultures from tumor tissue   specimens   - Response prediction and the microbiome in ESCC |
| Interim analyses | Not planned |
| Safety Data | - AEs, SAEs and treatment emergent adverse events according to CTC 4.03 - Frequency of clinically significant abnormal laboratory parameters |
| Sample size calculation | It is hypothesized that Nivolumab and Ipilimumab will increase overall survival. It is assumed that an immunotherapy approach consisting of a Nivolumab monotherapy in conjunction with a safety guided treatment escalation to a NIVO/IPI combination regimen increases the 1-year overall survival rate by a margin of 13% compared to historical control for standard chemotherapy (i.e. Nivolumab monotherapy followed by a conditional Nivolumab + Ipilimumab therapy 1-yr-OS = 30% vs CTx-control 1-yr-OS = 17%).  Sample size estimation:  Based on these assumptions, and an exponential shape of the survival curve, a one-sided, one-sample log rank test calculated from a sample of 69 subjects achieves 90.3% power at a alpha=0.05 one-sided significance level to detect a proportion surviving of 0.3 in the experimental group when the proportion surviving in the historic control group is 0.17. These proportions surviving are for a period of 12 month (1-year-OS rate). Subjects are accrued for a period of 12 month. Follow-up continues for a period of 24 month after the last subject is added. The probability that a subject experiences an event during the study is 0.9477. The expected number of events during the study is 65. To compensate for uninformative drop-outs a total of **N=75** subjects need to be recruited. |
| Study plan | First Patient In (FPI): Q4/2017  Last Patient In (LPI): after approx. 12 months  Last Patient Last treatment (LPLT): after approx. 20 months  End of follow-up period after LPI: after approx. 36 months  Study report: after approx. 45 months  Publication: after approx. 45 months |
